# The impact of antimicrobial resistance awareness interventions involving schoolchildren, development of an animation and parents engagements: a pilot study

**DOI:** 10.1186/s13756-022-01062-6

**Published:** 2022-02-04

**Authors:** Bernard Appiah, Lucy Asamoah-Akuoko, Elfreda Samman, Augustina Koduah, Irene Akwo Kretchy, Julius Yaw Ludu, Gloria Odonkor, Su Hyun Nam, Martha Gyansa-Luterrodt

**Affiliations:** 1 Research Program on Health Communication and Public Engagement (H-COPE), Department of Public Health, Falk College, Syracuse University, Syracuse, NY 13244 USA; 2 Centre for Science and Health Communication, PMB M71, Accra, Ghana; 3 National Blood Service Ghana, Research and Development, P.O. Box KB 78, Korle-Bu, Accra, Ghana; 4 Department of Health Promotion and Community Health Sciences, School of Public Health, Texas A&M University, College Station, TX USA; 5 Department of Pharmacy Practice and Clinical Pharmacy, School of Pharmacy, University of Ghana, Legon, Accra, Ghana; 6 Department of Computer Science, University of Ghana, Legon, Accra, Ghana; 7 Health Outcomes Division, College of Pharmacy, The University of Texas at Austin, Austin, TX USA; 8 Department of Transmedia, College of Visual and Performing Arts, Syracuse University, 100 Crouse Drive, Syracuse, NY 13244 USA; 9 Directorate of Pharmaceutical Services, Ministry of Health, Accra, Ghana

**Keywords:** Antimicrobial resistance, Parents, Schoolchildren, Animation, Public engagement

## Abstract

**Background:**

Antimicrobial resistance (AMR) is a global health challenge, particularly in low- and middle-income countries where antibiotics are widely available to consumers, leading to their misuse. However, AMR educational interventions for engaging parents of schoolchildren are mainly lacking in Sub-Saharan Africa. This study aimed to assess the potential of AMR animation and schoolchildren in influencing parents’ AMR knowledge, attitudes, and beliefs.

**Methods:**

Parents of schoolchildren aged 11–15 years in Tema, a city in Ghana, watched and discussed an AMR animation designed with ideas from the schoolchildren’s top stories and picture drawings. The children from two schools were first engaged with AMR lessons, with one school using storytelling, the other school using picture drawing, and none serving as a control. The children were then asked to discuss the lessons with their parents. Baseline surveys of parents of randomly selected children were conducted to assess AMR knowledge, attitudes and beliefs before engaging the students and parents, and immediately after the parents participated in viewing and discussing the animation. McNemar and t-tests were used to assess changes in AMR knowledge, attitudes and beliefs.

**Results:**

Parents who participated in the animation event, and whose schoolchildren were in the storytelling intervention school had significantly improved knowledge regarding the statement “Antibiotics will cure any infection” (*p* = 0.021, χ2 = 0.711; 88% vs 50%) between baseline and endline. However, these parents also had statistically significant decreased scores regarding the statement “Antibiotics do not kill our good bacteria” (*p* = 0.021, χ2 = 1.042; 71.4% vs 40%) between baseline and endline. There was no significant effect on any statement among parents whose children were in the picture drawing school. However, t-test results combining the statements as composite scores showed statistically significant difference in only the attitude construct among parents whose children participated in storytelling intervention (*p* = 0.043) or picture drawing intervention (*p* = 0.019). There were no statistically significant changes in knowledge and beliefs constructs.

**Conclusions:**

This study shows that interventions involving schoolchildren with parents engagements and AMR animation could influence parents’ AMR attitudes. The intervention could also positively or negatively impact parents’ AMR knowledge. Modifications of the interventions may be needed for tackling AMR.

**Supplementary Information:**

The online version contains supplementary material available at 10.1186/s13756-022-01062-6.

## Introduction

Antimicrobial resistance (AMR) is a global health challenge because of the inappropriate use of antibiotics. AMR could lead to about 10 million deaths annually and about US$100 trillion as a cumulative cost to the global economy by 2050, with low- and middle-income countries at an increased risk [1]. Given the threats of AMR, the World Health Assembly adopted a global action plan in 2015 to tackle this global health challenge [2]. During a special meeting of the United Nations General Assembly in 2016, African nations committed to addressing AMR [3]. The plan of action included a need to adopt a One Health approach by integrating animal and human health concerns regarding AMR.

In March 2017, the United Nations Secretary-General established the Inter-Agency Coordination Group (IACG) on Antimicrobial Resistance (AMR) to guide practical strategies for ensuring sustained effective actions at the global level to address AMR [4]. Subsequently, the IACG’s recommendation led to the establishment of the Global Leaders Group on Antimicrobial Resistance in January 2021 to strengthen global leadership on AMR, with members selected from UN member states including Nigeria and Senegal, the private sector, and civil society [5, 6]. 

Despite the level of resistance to prescribed antibiotics in Africa, a recent review found that current data on AMR are mainly lacking [7]. In Ghana, several studies including document analysis and key informant interviews of four policymakers [8], laboratory analyses of isolates of clinical specimens [9–13] and a cross-sectional study involving 379 prescribers [14] have identified AMR as a threat. These studies lack the perspectives of members of the public, including parents of schoolchildren.

The World Health Organisation Action Plan on Antimicrobial Resistance enumerates five strategic objectives. The first is a need to “[i]mprove awareness and understanding of antimicrobial resistance through effective communication, education and training” [2]. Such an awareness of AMR can be increased among key actors such as governments, healthcare workers, children and parents. A recent review that assessed interventions for influencing AMR behaviours such as using antibiotics to treat the common cold among the public identified 20 studies made up of controlled or non-controlled before-and-after studies, randomized or non-randomized controlled trials, cohort studies and interrupted time series studies [15]. Of the 20 studies reported, 19 were conducted in high-income countries, with a lone study from Moldova, an upper middle-income country. None was conducted in Africa.

According to the review, interventions aimed at schoolchildren and their parents could be key for addressing AMR given their effectiveness in improving knowledge, attitudes, and changing behaviours regarding responsible or rational use of antibiotics. For example, four of the six interventions that targeted parents positively influenced parents’ antimicrobial stewardship behaviour, with all the six interventions showing improved parents’ knowledge on AMR [15].

A more recent global survey of antibiotic awareness campaigns identified 60 projects in 16 low-and middle-income countries (LMICs) and 31 high-income countries, but only three were implemented in Africa [16]. Of the 25 (42%) campaigns that had evaluation data, 16 (27%) evaluated knowledge of the public. The survey also identified the difficulty of translating complex AMR messages to aid public understanding [16].

The use of digital communication technologies, including animation can not only be used to explain complex concepts of AMR, but can also be widely circulated more through social media channels. For example, an animation on AMR created with inputs from aquaculture farmers in Bangladesh was viewed on Facebook 17,614 times (Bangla version) and 11,200 Twitter times (English version) within the first 6 months [17].

Given the lack of studies on public awareness of AMR issues in Africa, particularly among parents of schoolchildren, there is a need to have interventions that can help address the complex topic of AMR in this region. This study assessed the effectiveness of an AMR animation intervention in influencing the knowledge, attitudes, and beliefs of parents of schoolchildren in Ghana. The study also assessed the potential influence of schoolchildren discussing AMR lessons learned in schools with their parents in influencing parents’ knowledge, attitudes and beliefs regarding AMR.

## Methods

### Study design

This study used ideas from schoolchildren to create an animation on AMR through a participatory design. It also used a prospective, single group randomized design to assess the effectiveness of animation, schoolchildren, and parent engagements in influencing beliefs, attitudes, and knowledge of parents of schoolchildren regarding AMR.

### Production of animation

Creating the animation involved several steps. First, two Junior High Schools for children aged 11–15 years were purposefully selected from the Tema metropolis of the Greater Accra Region of Ghana. Second, one school was purposively exposed to storytelling while the other school used picture drawing as AMR public engagement approaches. However, the schoolchildren and their parents who participated in the evaluation were randomly selected.

Given that the study was exploratory and did not have adequate clusters (only two schools) for cluster randomized controlled trial [18, 19], the two schools were not randomized to receive the interventions. For example, a simulation study suggests that detecting small effects in a study with even four clusters is difficult [18].

Third, for 8 weeks (September–November 2017), science teachers from each school taught the schoolchildren how to use picture drawing or storytelling to depict AMR messaging. Fourth, each school held a competition on February 28, 2018 to select either the top five stories or five pictures based on creativity and the messaging that reflects AMR. Details of the children’s involvement in the project, and the project’s impacts on AMR knowledge, attitudes and beliefs of the children have been published [20]. Fifth, an animator thoroughly assessed the 5 drawings from one school and the 5 stories from other school. The animator used the ideas from the drawings and the stories to create an animation. [see Additional file 1 for an example of a top story and a picture drawing from which the animation idea was created].

A 10-min animation was initially planned. However, based on discussions with the animator, and the need to devote more time to discuss the animation on AMR, the duration was shortened to 3 min. The animation was based on the Information, Motivation and Behavioral (IMB) skills model as a theoretical framework. According to the IMB skills model, well-informed (or knowledgeable) and well-motivated individuals with adequate skills for enacting behaviour will have better health behaviour [21]. The IMB skilld model has been applied mainly in the context of HIV interventions [21, 22], but has recently been used to explore determiants of rational drug use behaviour [23]. To the best of our knowledge, this is one of the first studies to use the IMB skills model to design an AMR animation in Sub-Saharan Africa. The information construct addresses the knowledge of parents and others who will watch the animation, the motivation construct addresses the attitudes while the behavioural skills construct is depicted by messages that encourage parents and others to get particular abilities to avoid or limit practices or behaviours that will lead to antimicrobial resistance.

### Field-testing and use of animation to engage parents and schoolchildren

On June 17, 2018, an event was held to field-test the animation, and to engage parents and schoolchildren of the two schools.

The event, which about 200 participants attended, involved showing the 3 min-animation, followed by a 2-h discussion. The 200 participants included parents who participated in the evaluation, and whose schoolchildren were randomly selected in each school for evaluating the impact of the interventions.

The discussion included questions about making the animation appealing and better reflecting AMR messaging. Some suggestions from the parents and students were integrated into the final animation. For example, the animation shown during the engagement meeting indicated someone throwing antibiotics into a bin or trash can. However, this was later changed because improperly discarding antibiotics could also lead to AMR. After edits, the final animation had a duration of 2 min 45 s [see Additional file 2]. The animator, who was also present at the event, and the implementation team discussed the need to change key aspects before finalizing the AMR animation.

During the engagement meeting with the parents and schoolchildren, there was a suggestion to also produce the animation in local languages to enable those who do not understand English, particularly residents of rural communities, to also benefit from it. However, the final animation is yet to be translated into local languages for engaging rural populations because of logistical challenges.

Schoolchildren aged 13–14 years and their parents were randomly selected from each of the two schools to evaluate the impact of the AMR animation. Using a single group exploratory study design involving two repeated measures (baseline and endline), alpha value of 0.05 and an effect size of 0.40 led to the sample being 26 children in storytelling intervention school and 26 children in the picture drawing intervention school [24]. The effect size of 0.40 was purposively selected as it was considered to be “large” enough for an exploratory study with no known previous indicators for guiding sample size calculation [24]. The sample size was increased to 31 in the storytelling and 32 in the picture drawing intervention to account for potential loss to follow-up.

Both the parents and their children were given an interviewer-administered baseline survey before science teachers engaged the students. However, the endline survey for the students occurred within a week after the competitions in the two schools. Unfortunately, their parents did not attend because the competitions occurred at a time when most were at work. This experience led to the implementation team selecting a weekend for the animation engagement activity with parents. The endline survey for the parents occurred immediately after viewing the animation and subsequently discussing it. This study focuses on only the impact on the parents, and not the impact on children, which has been published elsewhere [20]. The survey has 5 items on knowledge, 4 items on beliefs and 3 items on attitudes, and has been published [25], with answer options “Yes”, “No” or “Don’t Know”. The survey items were selected from a previous unpublished study on AMR involving parents and schoolchildren in Ghana that found the questions to be well understood. Trained interviewers who were not involved in the project’s implementation administered the questionnaire in English. However, translation of the scale in a local language (Twi) occurred in few instances when parents had a limited understanding of English. The interviewers practised the translations during training to ensure their accuracy.

In addition to answering questions on knowledge, attitudes and beliefs, the parents were asked to indicate whether their children had discussed issues on antimicrobial resistance and antibiotics with them in the past 6 months. This was to test the potential impact of word-of-mouth and animation as engagement approaches with parents of schoolchildren. Two datasets focusing on the parents’ responses are available [26].

### Data analysis

To quantify the effect of the interventions, the proportion of parents of schoolchildren representing each intervention school with correct responses to the 12 questionnaire items at baseline and endline were calculated. McNemar test was also conducted with IBM SPSS Statistics (version 22) to determine statistical significance between baseline and endline scores (within group analysis). Moreover, an independent t-test was conducted by combining the statements into knowledge, belief and attitude constructs, and determining the statistical significance between baseline and endline composite scores for parents of schoolchildren representing each intervention school (within group analysis). Ethical approval was obtained from research ethics committees of the Ghana Health Service (GHS-ERC-11/07/16) and Texas A&M University (IRB2016-0656D).

## Results

Out of the 31 parents whose children participated in the storytelling intervention, 25 participated in the endline survey. In contrast, out of the 32 parents whose children participated in the picture drawing intervention, 26 took the endline survey. Out of the 25 parents, most were female (72%) and were between the age of 38–47 years (56%). Out of the 26 parents whose children attended the picture drawing intervention school, most were female (78%), and 48% were aged 38–47 years.

At endline, 96% (24 out of 25) of the parents of children from the storytelling intervention school indicated that their children had discussed antimicrobial resistance and antibiotics issues with them whereas only 65.4% (17 out of 26) of parents of children in the picture drawing intervention school indicated so.

**Fig. 1:**
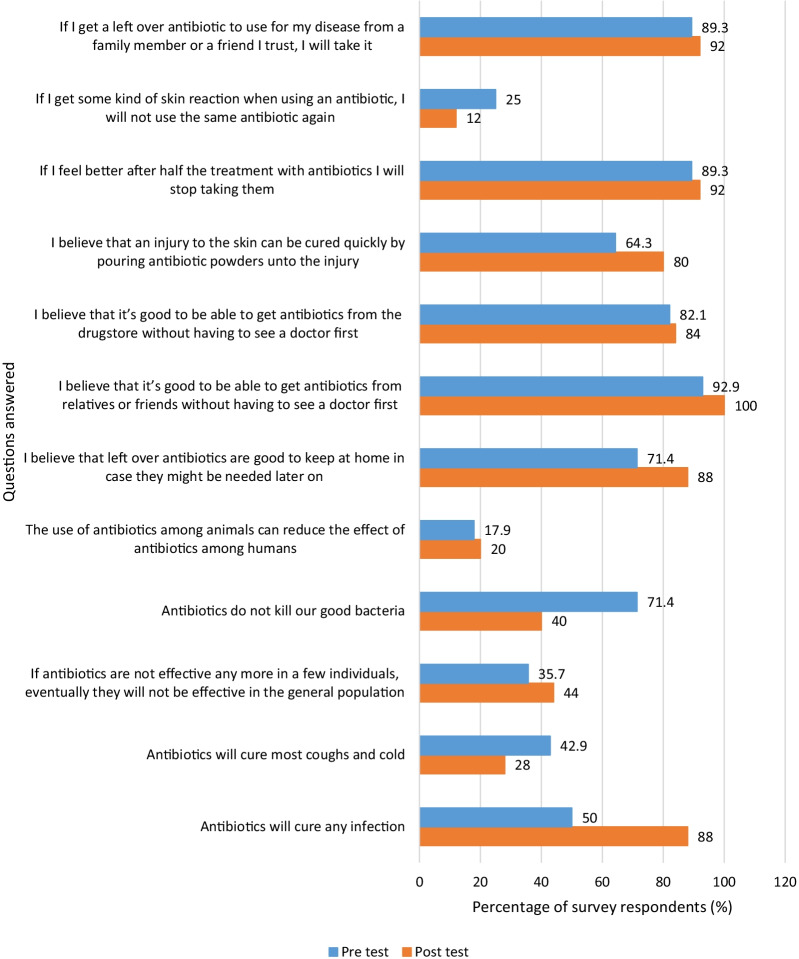
Percentage of parents of children in storytelling intervention school who answered questions correctly before and after the animation

Parents whose children participated in the storytelling intervention generally had improved endline scores compared with their baseline scores. (Fig. 1).

However, only one of the improved scores among parents whose schoolchildren were in the storytelling intervention school was statistically significant: “Antibiotics will cure any infection” (*p* = 0.021, χ2 = 0.711; 88% vs 50%) [Table 1]. Also, out of the three statements that had post-test scores being less than that of the pre-test scores, and thus showing a worse outcome, one was statistically significant: “Antibiotics do not kill our good bacteria” (*p* = 0.021, χ2 = 1.042; 71.4% vs 40%) (Table 1).

**Table 1:** Summary of survey responses for parents of schoolchildren in storytelling intervention school

Question	N	Pre and post responses^a^				McNemar *x*^2^	*p* value
		C&C	C&W	W&C	W&W		
*Knowledge*							
1. Antibiotics will cure any infection	25	13	1	9	2	0.711	0.021*
2. Antibiotics will cure most coughs and colds	25	4	7	3	11	0.682	0.344
3. If antibiotics are not effective anymore in a few individuals, eventually they will not be effective in the general population	25	3	6	8	8	0.649	0.791
4. Antibiotics do not kill our good bacteria	25	7	13	3	2	1.042	0.021*
5. The use of antibiotics among animals can reduce the effect of antibiotics among humans	25	2	2	3	18	2.679	1.000
*Beliefs*							
6. I believe that left-over antibiotics are good to keep at home in case they might be needed later on	25	15	2	7	1	0.003	0.180
7. I believe that it’s good to be able to get antibiotics from relatives or friends without having to see a doctor first	25	23	0	2	0	-	-
8. I believe that it’s good to be able to get antibiotics from the drugstore without having to see a doctor first	25	17	3	4	1	0.074	1.000
9. I believe that an injury to the skin can be cured quickly by pouring antibiotic powders onto the injury	25	14	1	6	4	4.167	0.125
*Attitudes*							
10. If I feel better after half the treatment with antibiotics, I will stop taking them	25	20	2	3	0	0.296	1.000
11. If I get some kind of skin reaction when using an antibiotic, I will not use the same antibiotic again	25	0	7	3	15	1.326	0.344
12. If I get a left-over antibiotic to use for my disease from a family member or friend I trust, I will take it	25	21	1	2	1	2.973	1.000

*Significant at 0.05 level

^a^The pre and post responses are written via symbols between the ‘&’ where C = correct, W = wrong. For example, C&C means correct pre level and correct post level answers and W&W means wrong pre level and wrong post level answers

**Fig. 2:**
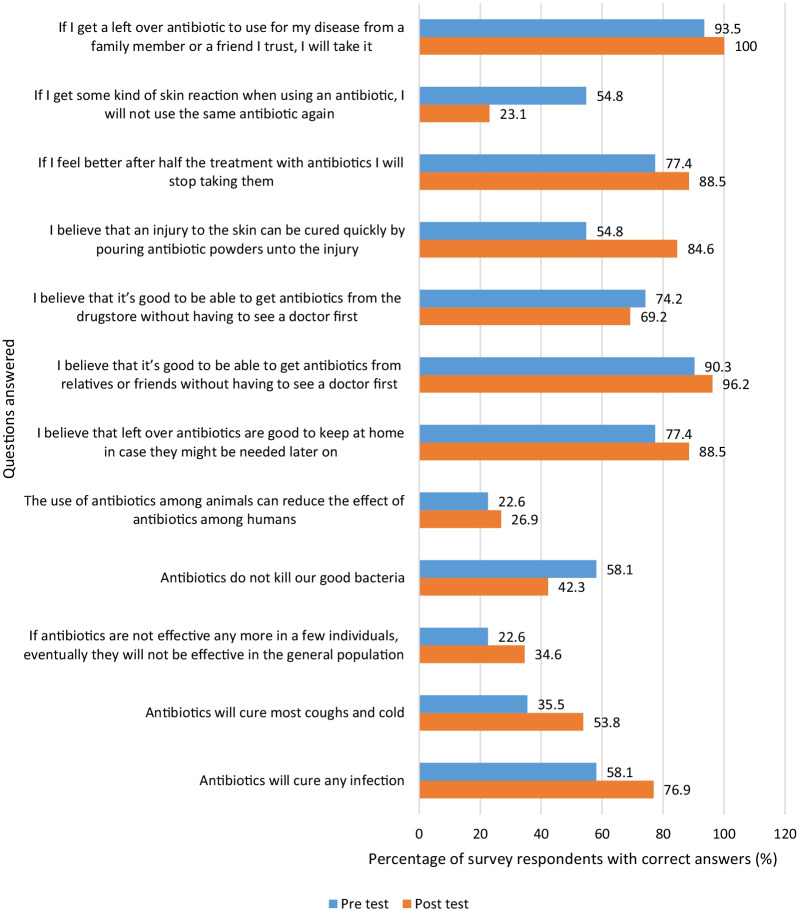
Percentage of parents of children in picture drawing intervention school who answered questions correctly before and after the animation

Similarly, among parents whose children were exposed to the picture drawing intervention and animation, 9 statements had improved correct scores at endline (Fig. 2).

However, unlike the parents of children exposed to the storytelling intervention and animation, none of the change in scores from baseline to endline for the parents of the picture drawing students was statistically significant (Table 2).

**Table 2:** Summary of survey responses for parents of schoolchildren in picture drawing intervention school

Question	N	Pre and post responses^a^				McNemar *x*^2^	*p* value
		C&C	C&W	W&C	W&W		
*Knowledge*							
1. Antibiotics will cure any infection	26	11	4	9	2	0.257	0.267
2. Antibiotics will cure most coughs and colds	26	6	4	8	8	0.248	0.388
3. If antibiotics are not effective any more in a few individuals, eventually they will not be effective in the general population	26	1	5	8	12	1.110	0.581
4. Antibiotics do not kill our good bacteria	26	4	10	7	5	2.345	0.629
5. The use of antibiotics among animals can reduce the effect of antibiotics among humans	26	2	5	5	14	0.013	1.000
*Beliefs*							
6. I believe that left-over antibiotics are good to keep at home in case they might be needed later on	26	18	2	5	1	0.201	0.453
7. I believe that it’s good to be able to get antibiotics from relatives or friends without having to see a doctor first	26	23	1	2	0	0.087	1.000
8. I believe that it’s good to be able to get antibiotics from the drugstore without having to see a doctor first	26	13	7	5	1	0.728	0.774
9. I believe that an injury to the skin can be cured quickly by pouring antibiotic powders onto the injury	26	12	3	10	1	0.580	0.092
*Attitudes*							
10. If I feel better after half the treatment with antibiotics, I will stop taking them	26	17	3	6	0	1.017	0.508
11. If I get some kind of skin reaction when using an antibiotic, I will not use the same antibiotic again	26	1	13	5	7	4.338	0.096
12. If I get a left-over antibiotic to use for my disease from a family member or friend I trust, I will take it	26	24	0	2	0	–	–

^a^The pre and post-test responses are written via symbols between the ‘&’ where C = correct, W = wrong. For example, C&C means correct pre level and correct post level answers and W&W means wrong pre level and wrong post level answers

Moreover, an independent t-test for parents whose children were in the storytelling intervention school indicated that there was no significant difference between baseline and endline with regard to knowledge construct (t(54) = 0.983, *p* = 0.330) and belief construct (t (54) = 0.615, *p* = 0.541). However, there was a significant difference between baseline and endline in the attitude construct [(t(31) = 2.110, *p* = 0.043)]. Similarly, regarding parents whose children were in the picture drawing intervention school, there was a significant difference between baseline and endline in the attitude construct [(t (36.8) = 2.449, *p* = 0.019)] but no significant difference between baseline and endline in the knowledge construct [(t(43.8) = 1.777, *p* = 0.083)] and belief construct [(t (36.5) = 1.432, *p* = 0.161)].

## Discussion

To the best of our knowledge, the current study presents the first empirical evidence from Sub-Saharan Africa for using AMR-themed animation created with ideas from schoolchildren, and the schoolchildren’s word-of-mouth as engagement approaches for influencing the knowledge, beliefs and attitudes of their parents regarding AMR. It is interesting to find that awareness interventions involving student and parent engagement and development of an animation had significant effects on parents’ knowledge.

The significant impact on knowledge but not beliefs and attitudes about antibiotics and AMR of parents in the storytelling intervention school exposed to the animation could be because the children in the storytelling intervention might have discussed the stories more with their parents than their counterparts in the picture drawing intervention. Schoolchildren in Junior High Schools of Ghana tend to write stories as part of their lessons, and discuss them with their parents. Thus, it was not surprising that 96% of parents of children at the storytelling intervention school indicated that their children discussed antibiotics with them. Only about 65% of parents with children at the picture drawing intervention school indicated that their children discussed antibiotics with them.

Moreover, storytelling offers a unique African tradition [27] that might have resonated well with parents of schoolchildren who participated in the storytelling intervention. However, it was also interesting to find t-test results indicating that the interventions had significant effects on parents attitudes but not knowledge and beliefs about AMR for those whose schoolchildren participated in either storytelling or picture drawing. This result suggests that the interventions could improve motivation—as depicted by the IMB skills model—to enable them to engage in behaviours such as avoiding the misuse of antibiotics that could prevent the development of AMR.

The study’s findings contribute to the literature on AMR interventions with parents as the priority populations. For example, an intervention on rational use of antibiotics that was implemented in the United States among childcare centre staff did not identify significant differences in knowledge scores among parents with no college education (*p* = 0.11) [28]. Thus, our finding that there was no significant effect of the interventions on composite knowledge score mirrors the United States study [28].

In Italy, an educational intervention that was implemented to address antimicrobial resistance issues in the general population found only one item on knowledge being statistically significant—“antibiotics are effective against viruses”—with some worse outcomes in both the intervention and control areas after the campaign [29]. Thus, our finding that the proportion of parents of schoolchildren exposed to the storytelling intervention, animation and discussion with the correct answer to the statement “Antibiotics do not kill our good bacteria” being worse at endline resembles the study in Italy [29]. However, unlike our study, the Italy study was an information campaign delivered by general practitioners and paediatricians.

The significantly positive effect on knowledge (“antibiotics will cure any infection”) among parents whose schoolchildren were exposed to storytelling regarding the knowledge statement that “Antibiotics will cure any infection” offers opportunities for future interventions. For example, with this finding, researchers will be able to perform the necessary power analysis for future quasi-experimental or cluster-randomized controlled trials to test the educational interventions on AMR that target the knowledge of parents. In this regard, our findings could contribute to future intervention development on AMR in Ghana among parents of schoolchildren.

Such future studies may need to test not only knowledge, attitudes and beliefs but also behaviours such as sharing antibiotics with others or use of antibiotics for treating common cold. For example a study involving schoolchildren of ages 12–13 years in Moldova in 21 intervention and 20 control schools was found to be successful at positively influencing behaviour: parents’ use of antibiotics for colds and flu [30]. In the current study, schoolchildren were asked to speak with their parents about AMR lessons they learned and parents were expected to attend the children’s competitions. However, the parents not attending the children’s competition event but attending only the animation viewing and discussion likely reduced the dosage of the intervention compared with the study in Moldova.

Given that the study involved only two schools because of its focus on generating preliminary data, randomization into intervention and control clusters did not occur unlike several studies that have explored AMR outside sub-Saharan Africa [29, 30]. However, the current study explores potential measures that have not been widely studied in AMR awareness elsewhere. For example, the statements “I believe that it’s good to be able to get antibiotics from relatives or friends without having to see a doctor first,” and “I believe that it’s good to be able to get antibiotics from the drugstore without having to see a doctor first” seem more applicable to low- and middle-income countries such as those in Sub-Saharan Africa where antibiotics can easily be obtained from drugstores without prescriptions. Thus, the study offers opportunities for other scholars to adapt the questionnaire for use in Ghana and other African settings.

Our study has implications for future AMR awareness interventions involving student and parent engagement and the development of an animation to influence parents of schoolchildren. There is a need to require that schoolchildren send their drawings home and discuss them with their parents to make the use of picture drawing an effective engagement approach to positively influence the AMR knowledge, attitudes and beliefs of their parents. Also, if schoolchildren are expected to discuss antimicrobial resistance issues with their parents as part of school-based public engagement project, an objective assessment approach such as using home assignments that may require parental signature. However, a challenge with this approach in Ghana and other low-resource settings may be that some parents may not be literate, thus making it difficult for them to sign assignments. For example, about 35% of the adult population in Sub-Saharan Africa cannot read or write [31].

Moreover, this study identified key lessons when implementing such engagement projects with parents of schoolchildren. For example, the evaluation team used Parents Teachers Associations (PTAs) of both schools to collect baseline data on parents whose children were randomly selected to participate in the evaluation. The implementation team expected the PTAs of both schools to organise a meeting each to facilitate final engagement with them. However, the PTAs of both schools did not have a regular schedule for this to occur. Because of such a challenge, the implementation team devised an alternative strategy in consultation with the project’s technical advisory committee. This led to an agreement to host the event for both schools on a Sunday afternoon after church. Both parents and children successfully attended this event, but it also limited the number of parents and children expected to be engaged with the animation. The Sunday event for the parents and children from the two schools also made it challenging to collect data from parents because of the limited time. In sum, using the PTA meetings worked well as a platform to collect baseline data among parents, but not as an engagement platform. Organising an engagement meeting on a Sunday after church was hugely successful for both parents and children. Scholars or practitioners need not rely mainly on PTA meeting times to actively involve parents in public engagement AMR projects.

Overall, the study have implications for designing, implementing and evaluation behavioural change interventions related to AMR. For example, such interventions need to consider cultural factors [32] and evidence-based theories [33] to guide their development, implementation and evaluation. The type of theories to be selected would depend on the direction of socio-behavioral change expected. For example, behavioral theories can be categorised according to whether they are applicable to behaviours that should be decreased, increased or both [33]. The IMB skills model can be used to study behaviours that should be increased to tackle AMR (for example, using antibiotics for only bacterial infections) or behaviours that should be decreased to tackle AMR (for example, sharing antibiotics with friends and relatives or using antibiotics to treat the common cold).

In evaluating theory-based AMR interventions that aim to change AMR behaviour, implementation science measures such as feasibility, acceptability and fidelity would be critical [34]. In this context, the current study provides an initial evidence for the feasibility of using AMR awareness interventions involving schoolchildren, parent engagement and development of an animation.

However, despite the strengths of the findings of our study, it has some limitations. It is unclear whether the effects observed were due to the animation alone, word-of-mouth from the children or both. Thus, future studies would need to consider quantifying separate effects. Such effects can be determined through robust designs such as cluster randomised controlled trials.

Moreover, the project relied on trained interviewer-administered questionnaire surveys. Such surveys have the potential of interviewer bias and respondents giving socially desirable answers. To minimise potential interviewer bias, the data collectors were not part of the implementation team, and were adequately trained.

## Conclusion

This study showed that using an animation on AMR designed with ideas from schoolchildren could influence parents’ AMR knowledge and attitudes. However, the effect could be positive or negative. When designing school-based AMR educational interventions that aim to engage parents of schoolchildren, public engagement researchers and practitioners would need to consider identifying strategies for encouraging the children to discuss the lessons effectively with their parents. To the best of our knowledge, our study provides the first evidence regarding the use of antimicrobial resistance awareness interventions involving student and parent engagement and the development of an animation to influence AMR knowledge, beliefs and attitudes of parents of schoolchildren in Sub-Saharan Africa.

## Supplementary Information

**Additional file 1.** An antimicrobial resistance animation created with ideas from schoolchildren.

**Additional file 2.** A sample each of story telling and picture drawing from which the animation was created.

## Data Availability

All data created and analysed for this study are included in this published article.

## Abbreviations

AMR: Antimicrobial resistance
PTA: Parent Teacher Association
IACG: Inter-Agency Coordination Group

## References

[1] O’Neill J. Tackling drug-resistant infections globally: final report and recommendations; 2016. http://amr-review.org/sites/default/files/160525_Final%20paper_with%20cover.pdf. Accessed July 1 2021.

[2] World Health Organization Global action plan on antimicrobial resistance [homepage on the Internet]; 2015. http://apps.who.int/iris/bitstream/10665/193736/1/9789241509763_eng.pdf. Accessed July 1 2021.

[3] United Nations Political declaration of the high-level meeting of the General Assembly on antimicrobial resistance (A/RES/71/3) [homepage on the Internet]. https://digitallibrary.un.org/record/845917/files/A_RES_71_3-EN.pdf. Accessed July 22 2021.

[4] WHO Future Global Governance for Antimicrobial Resistance. IACG Discussion Paper; 2018. https://www.who.int/antimicrobial-resistance/interagency-coordination-group/IACG_Future_global_governance_for_AMR_120718.pdf. Accessed July 22 2021.

[5] UN FAO. Prominent global leaders in science, industry and government meet to step up fight against antimicrobial resistance; 2021. http://www.fao.org/news/story/en/item/1371017/icode/. Accessed July 3 2021.

[6] WHO. Global Leaders Group on Antimicrobial Resistance; 2021. https://www.who.int/groups/one-health-global-leaders-group-on-antimicrobial-resistance. Accessed July 25 2021.

[7] Tadesse BT, Ashley EA, Ongarello S, Havumaki J, Wijegoonewardena M, González IJ, Dittrich S (2017). Antimicrobial resistance in Africa: a systematic review. BMC Infect Dis.

[8] Yevutsey SK, Buabeng KO, Aikins M, Anto BP, Biritwum RB, Frimodt-Møller N, Gyansa-Lutterodt M (2017). Situational analysis of antibiotic use and resistance in Ghana: policy and regulation. BMC Public Health.

[9] Newman MJ, Frimpong E, Donkor ES, Opintan JA, Asamoah-Adu A (2011). Resistance to antimicrobial drugs in Ghana. Infect Drug Resist.

[10] Opintan JA, Newman MJ, Arhin RE, Donkor ES, Gyansa-Lutterodt M, Mills-Pappoe W (2015). Laboratory-based nationwide surveillance of antimicrobial resistance in Ghana. Infect Drug Resist.

[11] Feglo PK, Gbedema SY, Quay SN, Adu-Sarkodie Y, Opoku-Okrah C (2010). Occurrence, species distribution and antibiotic resistance of Proteus isolates: a case study at the Komfo Anokye Teaching Hospital (KATH) in Ghana. Int J Pharm Sci Res.

[12] Saba CK, Amenyona JK, Kpordze SW (2017). Prevalence and pattern of antibiotic resistance of *Staphylococcus aureus* isolated from door handles and other points of contact in public hospitals in Ghana. Antimicrob Resist Infect Control.

[13] Dekker D, Wolters M, Mertens E, Boahen KG, Krumkamp R, Eibach D, Schwarz NG, Adu-Sarkodie Y, Rohde H, Christner M, Marks F (2016). Antibiotic resistance and clonal diversity of invasive *Staphylococcus aureus* in the rural Ashanti Region, Ghana. BMC Infect Dis.

[14] Asante KP, Boamah EA, Abdulai MA, Buabeng KO, Mahama E, Dzabeng F, Gavor E, Annan EA, Owusu-Agyei S, Gyansa-Lutterodt M (2017). Knowledge of antibiotic resistance and antibiotic prescription practices among prescribers in the Brong Ahafo Region of Ghana; a cross-sectional study. BMC Health Serv Res.

[15] Price L, Gozdzielewska L, Young M, Smith F, MacDonald J, McParland J, Williams L, Langdridge D, Davis M, Flowers P (2018). Effectiveness of interventions to improve the public’s antimicrobial resistance awareness and behaviours associated with prudent use of antimicrobials: a systematic review. J Antimicrob Chemother.

[16] Huttner B, Saam M, Moja L, Mah K, Sprenger M, Harbarth S, Magrini N (2019). How to improve antibiotic awareness campaigns: findings of a WHO global survey. BMJ Glob Health.

[17] Thornber K, Huso D, Rahman MM, Biswas H, Rahman MH, Brum E, Tyler CR (2019). Raising awareness of antimicrobial resistance in rural aquaculture practice in Bangladesh through digital communications: a pilot study. Glob Health Action.

[18] Deke J (2016). Design and analysis considerations for cluster randomized controlled trials that have a small number of clusters. Eval Rev.

[19] Goesling B (2019). A practical guide to cluster randomized trials in school health research. J Sch Health.

[20] Appiah B, Anum-Hagin D, Gyansa-Luterrodt M, Samman E, Agyeman FK, Appiah G, Odonkor G, Ludu JY, Osafo J, Rene A (2021). Children against antibiotics misuse and antimicrobial resistance: assessing effectiveness of storytelling and picture drawing as public engagement approaches. Wellcome Open Res.

[21] Fisher JD, Fisher WA, Misovich SJ, Kimble DL, Malloy TE (1996). Changing AIDS risk behavior: effects of an intervention emphasizing AIDS risk reduction information, motivation, and behavioral skills in a college student population. Health Psychol.

[22] Cornman DH, Kiene SM, Christie S, Fisher WA, Shuper PA, Pillay S, Friedland GH, Thomas CM, Lodge L, Fisher JD (2008). Clinic-based intervention reduces unprotected sexual behavior among HIV-infected patients in KwaZulu-Natal, South Africa: results of a pilot study. J Acquir Immune Defic Syndr (1999).

[23] Bian C, Xu S, Wang H, Li N, Wu J, Zhao Y, Li P, Lu H (2015). A study on the application of the information-motivation-behavioral skills (IMB) model on rational drug use behavior among second-level hospital outpatients in Anhui, China. PloS one..

[24] Barcikowski RS, Robey RR. Sample size selection in single group repeated measures analysis. In: Paper presented at the annual meeting of the American Educational Research Association, Chicago, IL. Washington, DC: American Educational Research Association; 1985.

[25] Appiah B. Correct answers to quantitative questions; 2021. 10.6084/m9.figshare.14207006.

[26] Appiah B. Parents data on engagement with antimicrobial resistance animation in Ghana; 2021. 10.7910/DVN/L3YMBN.

[27] Kwakye-Opong R, Gharbin E (2017). The Atwa Kodzidan: a unique African Storytelling Theatre tradition and architecture in Ghana. Africology J Pan Afr Stud.

[28] Croft DR, Knobloch MJ, Chyou P, Elzen DV, Janette C, Davis JP, Besser RE, Belongia EA (2007). Impact of a child care educational intervention on parent knowledge about appropriate antibiotic use. WMJ-MADISON-.

[29] Formoso G, Paltrinieri B, Marata AM (2013). Feasibility and effectiveness of a low cost campaign on antibiotic prescribing in Italy: community level, controlled, non-randomised trial. BMJ.

[30] Cebotarenco N, Bush PJ (2008). Reducing antibiotics for colds and flu: a student-taught program. Health Educ Res.

[31] UNESCO. Fact Sheet No. 45; 2017 http://uis.unesco.org/sites/default/files/documents/fs45-literacy-rates-continuerisegeneration-to-next-en-2017.pdf.

[32] Ayukekbong JA, Ntemgwa M, Atabe AN (2017). The threat of antimicrobial resistance in developing countries: causes and control strategies. Antimicrob Resist Infect Control.

[33] Patey AM, Hurt CS, Grimshaw JM, Francis JJ (2018). Changing behaviour ‘more or less’—do theories of behaviour inform strategies for implementation and de-implementation? A critical interpretive synthesis. Implement Sci.

[34] Weiner BJ, Lewis CC, Stanick C, Powell BJ, Dorsey CN, Clary AS, Boynton MH, Halko H (2017). Psychometric assessment of three newly developed implementation outcome measures. Implement Sci.

