# Supplementary material for: The impact of antimicrobial resistance awareness interventions involving schoolchildren, development of an animation and parents engagements: a pilot study
Source: Antimicrob Resist Infect Control. 2022 Feb 4;11:26. doi: 10.1186/s13756-022-01062-6 (PMC8817549; doi:10.1186/s13756-022-01062-6)
Supplement: Supplementary file 1 — Additional file 1. An antimicrobial resistance animation created with ideas from schoolchildren. [file 13756_2022_1062_MOESM1_ESM.docx]

Additional file 1

An antimicrobial resistance animation created with ideas from schoolchildren

https://www.youtube.com/watch?v=BVTh-BieBIo
