# Supplementary material for: The impact of antimicrobial resistance awareness interventions involving schoolchildren, development of an animation and parents engagements: a pilot study
Source: Antimicrob Resist Infect Control. 2022 Feb 4;11:26. doi: 10.1186/s13756-022-01062-6 (PMC8817549; doi:10.1186/s13756-022-01062-6)
Supplement: Supplementary file 2 — Additional file 2. A sample each of story telling and picture drawing from which the animation was created. [file 13756_2022_1062_MOESM2_ESM.docx]

**A sample each of story telling and picture drawing from which the animation was created**

**Story telling: A sample**

**THE PEOPLE OF ASSENSO AND THE ANTIBIOTIC EXPERIENCE**

In the community where I lived, there was a chronic outbreak of cold, cough and flu. The community did not know what to do so I took the initiative and ran to the community hospital for aid. The nurses came rushing with stretchers to convey the people to the hospital to be attended to urgently. After they had been diagnosed by the health workers, the doctors realised that the diseases were caused by microbes and bacteria and so they prescribed some antibiotics amoxicillin, keflex, flagyl and tetracycline hydrochloride basutopan for the patients and later discharged them. Subsequently after some few months, the people noticed signs of the same illness that infected them. This was because of the over use of the drugs by the villagers which resulted in making the bacteria become immune to the antibiotics. The bacteria had altered the permeability of their membrane to prevent the antibiotic drugs from reaching its target to halt their growth. This made the community members severely ill such that some doctors had to travel from afar to the village to educate the villager on the negative effects of abusing the antibiotics to cure such illness when basic home remedies could be used instead. The invitation of these doctors and their sensitization or orientation given to us, brought my community back to its usual state.

*Message:*

Avoid taking antibiotics to cure flu and common colds since these infections tend to be viral and cannot be cured with only these antibiotics. This is called the Antimicrobial Resistance.

**Picture drawing: A sample**

**
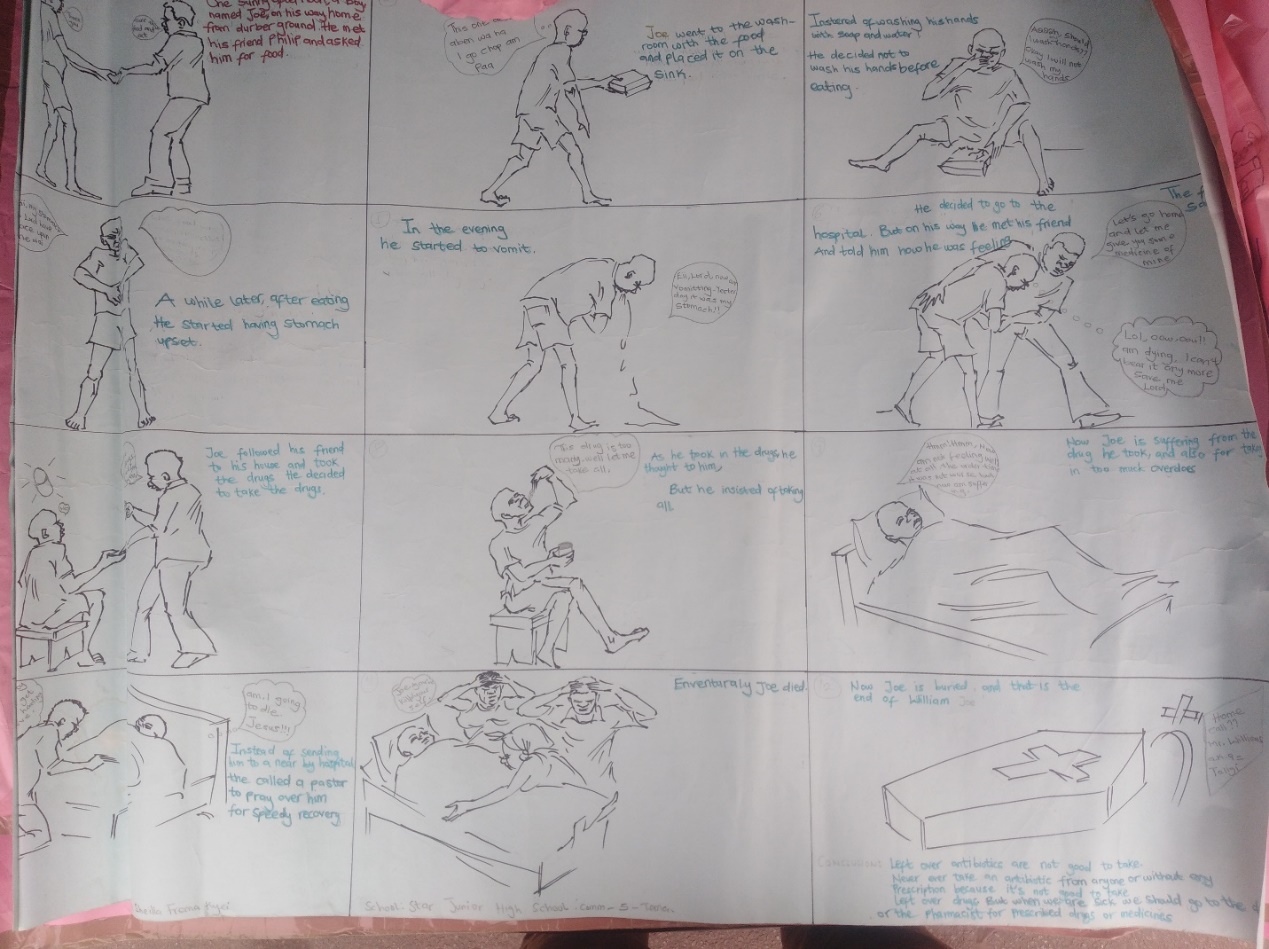
**
